# Characterizing Body Image Distortion and Bodily Self-Plasticity in Anorexia Nervosa via Visuo-Tactile Stimulation in Virtual Reality

**DOI:** 10.3390/jcm9010098

**Published:** 2019-12-30

**Authors:** Luca Provenzano, Giuseppina Porciello, Sofia Ciccarone, Bigna Lenggenhager, Gaetano Tieri, Matteo Marucci, Federico Dazzi, Camillo Loriedo, Ilaria Bufalari

**Affiliations:** 1 Department of Psychology, “Sapienza” University of Rome, 00185 Rome, Italy; luca.provenzano@uniroma1.it (L.P.); sofia.ciccarone@uniroma1.it (S.C.); matteo.marucci@uniroma1.it (M.M.); 2 IRCCS, Santa Lucia Foundation, 00142 Rome, Italy; gaetano.tieri@gmail.com; 3 Department of Psychology, University of Zurich, 8050 Zurich, Switzerland; bigna.lenggenhager@gmail.com; 4 Virtual Reality Lab, University of Rome Unitelma-Sapienza, 00161 Rome, Italy; 5 BrainTrends Ltd., Applied Neuroscience, Gallicano nel Lazio (RM), 00010 Rome, Italy; 6 Department of Human Sciences, Lumsa University, 00193 Rome, Italy; federicodazzi@hotmail.com; 7 Clinica Psichiatrica, “Sapienza” University of Rome, 00185 Rome, Italy; camillo.loriedo@uniroma1.it; 8 Department of Developmental and Social Psychology, “Sapienza” University of Rome, 00185 Rome, Italy

**Keywords:** anorexia nervosa, body image distortion, body dissatisfaction, embodiment, virtual reality, interpersonal multisensory stimulation

## Abstract

We combined virtual reality and multisensory bodily illusion with the aim to characterize and reduce the perceptual (body overestimation) and the cognitive-emotional (body dissatisfaction) components of body image distortion (BID) in anorexia nervosa (AN). For each participant (20 anorexics, 20 healthy controls) we built personalized avatars that reproduced their own body size, shape, and verisimilar increases and losses of their original weight. Body overestimation and dissatisfaction were measured by asking participants to choose the avatar that best resembled their real and ideal body. Results show higher body dissatisfaction in AN, caused by the desire of a thinner body, and no body-size overestimation. Interpersonal multisensory stimulation (IMS) was then applied on the avatar reproducing participant’s perceived body, and on the two avatars which reproduced increases and losses of 15% of it, all presented with a first-person perspective (1PP). Embodiment was stronger after synchronous IMS in both groups, but did not reduce BID in participants with AN. Interestingly, anorexics reported more negative emotions after embodying the fattest avatar, which scaled with symptoms severity. Overall, our findings suggest that the cognitive-emotional, more than the perceptual component of BID is severely altered in AN and that perspective (1PP vs. 3PP) from which a body is evaluated may play a crucial role. Future research and clinical trials might take advantage of virtual reality to reduce the emotional distress related to body dissatisfaction.

## 1. Introduction

Anorexia nervosa (AN) affects mostly adolescent and young women [1], has the highest mortality rate among all psychiatric disorders [2] and is largely resistant to currently available treatments [3]. A core clinical symptom of AN is body image distortion (BID), which impacts onset, prognosis, and relapse [4]. Body image is a multifaceted construct that comprises body-related perception, expectations, thoughts, feelings, and actions [5,6] which are represented in dedicated neural circuitries [7,8,9,10]. In AN, BID affects both perceptual and cognitive-emotional components of the body representation [11], i.e., patients typically overestimate their body size [12] and report higher body dissatisfaction [13] than healthy controls (HC) [14].

The use of new sophisticated and biometrically plausible distortion methods, made possible by immersive virtual reality contexts, has paved the way for precisely measuring body overestimation e.g., [15], whereas the development of interpersonal multisensory stimulation (IMS) paradigms has increased insights into the plasticity of the bodily self [15,16]. In IMS paradigms, participants typically experience a tactile stimulation on their own body synchronously with an observed touch at the corresponding body part on another individual’s body [17,18,19] which leads to the illusory sensation of ownership toward the latter (termed embodiment), as evidenced by subjective, behavioral, and physiological measures [20,21,22,23,24].

IMS paradigms have been extended to virtual avatars observed from a first-person perspective (1PP) [25], even of different sizes [26], which might lead to a change in one’s own body perception according to the avatar size [27]. Specifically, in HC identification with a slim virtual body reduces not only the overestimation of the own body size but also increases body satisfaction [28], while identification with an obese avatar induces body dissatisfaction [28].

Embodiment illusions might thus represent a promising tool to reduce BID in AN. Preliminary results in this field suggest that, in AN patients, illusory ownership of a fake hand is enhanced [29] and leads to a reduction in hand-size overestimation [30]. Also, embodying a normal body mass index (BMI) avatar reduces overestimation of shoulders, abdomen, and hips [31]. However, this effect occurs after both synchronous (experimental condition) and asynchronous (control condition) IMS, suggesting that it might not be due to the embodiment per se, but rather to purely visual effects.

Thus, the few existing studies using embodiment illusions in AN patients tentatively suggest that a normalization of BID is possible. Yet, it is still unclear how robust the effect is, to what degree it is linked to embodiment per se and how such illusions affect and interact with the affective-emotional components of body representation, which are central to BID [11,32].

Here, we addressed these issues by: (i) individualizing the avatars for each of our participant’ body (unlike previous studies); (ii) assessing the embodiment strength both at the explicit (questionnaires, e.g., [19,33]) and implicit level (body temperature, e.g., [34], but see [35] for a critical account), and (iii) measuring both perceptual and emotional aspects of BID before and after the embodiment of three different sized avatars was induced. We tested young females with AN and low-BMI age-matched HC with no diagnosis of eating disorders.

We expected AN patients to overestimate their body size [12] and to show higher body dissatisfaction than HC [13], as indexed by clinical measures (clinical questionnaires’ scores) and by the higher discrepancy between one’s own perceived and ideal body [36]. Furthermore, according to Eshkevari and colleagues [29], we expected higher bodily self-plasticity (namely higher embodiment measured both at the implicit and explicit level) in AN patients compared to HC. Importantly, we hypothesized that body dissatisfaction would decrease in AN patients as an effect of embodying a body which corresponds or is thinner than the perceived one [28]. Lastly, we expected that embodying an avatar larger than the perceived one would enhance negative emotions in AN patients more than HC.

## 2. Materials and Methods

### 2.1. Participants

A total of 21 female patients diagnosed with AN and 22 age-matched HC were recruited. All AN patients were diagnosed with anorexia nervosa (restricting type) by the Department of Psychiatry and Eating Disorder of the Hospital Policlinico Umberto I, which followed the criteria of Diagnostic and Statistical Manual of Mental Disorder—5 [37]. One AN patient was later excluded because of diagnostic migration, i.e., the diagnosis changed from AN to major depression as primary disorder with a secondary eating disorder component. Two HC were excluded for technical problems. A total of 20 AN patients ((mean ± standard error (SE)) (age = 23.30 ± 7.61, BMI = 15.87 ± 1.12)) and 20 HC (age = 23.85 ± 3.23, BMI = 18.98 ± 1.01) finally participated in the study. For the HC, the presence and/or history of any eating disorder and/or other psychiatric disorders constituted an exclusion criterion, whereas a BMI score in the lower normal range (i.e., between 17 and 21) was an inclusion criterion. The study was approved by the Ethical Committees of Policlinico Umberto I and IRCCS Santa Lucia Foundation and in accordance with the ethical standards of the 2013 Declaration of Helsinki. All the participants read and signed the informed consent.

### 2.2. Procedure

The experiment consisted of two sessions: a pre-experimental (Section 2.3) and an experimental session (Section 2.4), with about one week break in between, in which the individualized avatars were created.

### 2.3. Pre-Experimental Session

This session lasted about one hour. Participants filled out a series of questionnaires presented in randomized order on a computer using E-Prime^®^ 2.0 software. The Eating Disorder Inventory—2 (EDI-2) [38], the Body Shape Questionnaire (BSQ) [39], the Body Uneasiness Test (BUT) [40], and the Bulimic Investigatory Test, Edinburgh (BITE) [41] were used to assess the symptoms severity of the eating disorder pathology, whereas the Symptom Checklist-90-R (SCL-90-R) [42] was used to check for the presence of others psychiatric symptoms (see Supplementary Materials for detailed information).

Subsequently, a female experimenter measured circumferences and lengths of selected body parts of each participants and took pictures of participants’ body standing up (front, back, and profile view) with a Nikon D40 mounted on a tripod. Participants’ pictures and body measures served to create the avatars personalized for each participant.

#### Avatars Modelling

**Figure 1:**
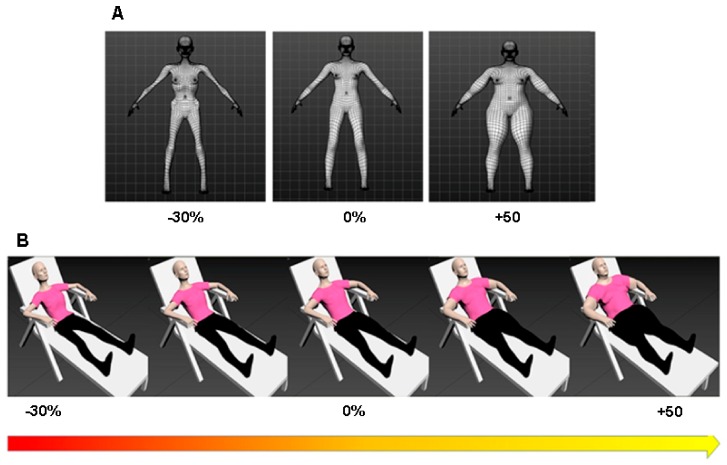
Creation of 3D Stimuli. (**A**) Example of three customized avatars built according to the participant’s body measures and pictures: an avatar that reproduced participant’s real body (avatar 0%), a thinner avatar (avatar −30%), and fatter avatar (+50%). (**B**) Example of avatars selection extracted from the continuum of avatars lying on a deck chair and increasing in size in steps of 3%, starting from the thinnest (−30%) to the fattest avatar (+50%).

A 3D modelling software (MakeHumans, open source tool for making 3d characters) was used to recreate the personalized avatar that matched participants’ real body in terms of height, shape, and body size and two more avatars that reproduced verisimilar loss of 30% and gain of 50% of the original weight (Figure 1, panel A). Specifically, Adobe Photoshop 7.0 (Adobe Systems Incorporated, San Jose, CA, USA) was used to create highly detailed skin, clothes, and material textures. Subsequently, these three avatars were imported into 3dsMax (Autodesk Inc., Mill Valley, CA), a 3D modelling and animation software, which we used to create a continuum of 28 avatars incrementing in size in steps of 3%, starting from the thinnest (−30%) to the fattest avatar (+50%). One set of 28 standing avatars facing the participants was created for subsequent task, i.e., the Avatar selection task (please see Section 2.4.1) in which participants could choose the avatar that best resembled their own body by observing it from a 3PP). We decided to present a set of avatars going from −30% to +50% of the original body size in order to not end up with unrealistically thin bodies (especially in the case of the AN) and to be able to measure the presence of body overestimation in the range suggested by a recent meta-analysis [12]. Another set of avatars was created lying on a deck chair (Figure 1, panel B) and used for inducing the embodiment (please see Section 2.4.3).

### 2.4. Experimental Session

**Figure 2:**
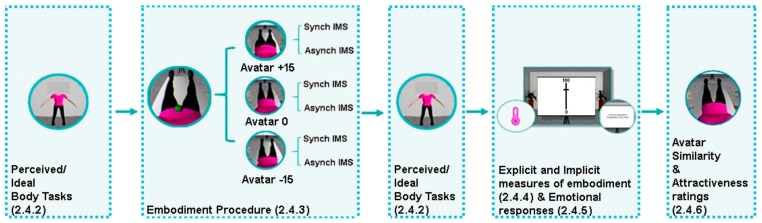
Experimental procedure. After selecting the avatar most similar to their perceived body and the one most resembling their ideal body, participants were enrolled in embodiment blocks in which synchronous and asynchronous interpersonal multisensory stimulation (IMS) were applied to three different bodies (the perceived body, −15% thinner body, +15% fatter body). After each embodiment block participants repeated the perceived and ideal body tasks to measure the effects of the embodiment of different sized avatars on body dissatisfaction. Explicit and implicit measures of the embodiment illusion, as well as the emotional response after being exposed to a/synchronous touching of different sized avatars were recorded after each embodiment block. At the end of the experiment we asked participants to rate from a first-person perspective the three avatars in terms of similarity to their own body and overall attractiveness.

This session lasted about two hours (see Figure 2 for an illustration of the procedure). Participants first performed the Avatar selection task (Section 2.4.1). Then they put on clothes that matched the avatar’s outfit and lay down on the deck chair to perform the perceived and ideal body tasks (Section 2.4.2). Afterward, participants experienced synchronous and asynchronous IMS (Section 2.4.3) with three different body size avatars (Avatar 0%., i.e., the avatar they chose in the Avatar selection task; Avatar −15% and Avatar +15%, i.e., an avatar 15% thinner and one 15% fatter than the one reproducing their own perceived body) in separate runs, counterbalanced across participants. Within each run participants received synchronous and asynchronous IMS in separate blocks (counterbalanced across participants) with the same avatar size. Immediately after each IMS block, participants performed the perceived body task (first 6 blocks) or the ideal body task (second 6 blocks, or vice versa). Then, we collected explicit and implicit measurements of embodiment (Section 2.4.4) and the emotional response (Section 2.4.5) to the IMS. At the end participants were also asked to rate the avatars in terms of similarity and attractiveness (Section 2.4.6).

All the experimental tasks were done in a virtual scenario that reproduced the actual experimental room, i.e., a 5 × 8 meters furnished room with a deck chair, identical to the one participant sat on during the experiment, and a 1.5 × 1.5 meters screen where questionnaires were projected.

#### 2.4.1. Avatar Selection Task

Participants were asked to choose the avatar that best fits their own body from a continuum ranging from a body that was 30% thinner to another one that was +50% fatter than the actual body, i.e., the body that was reproduced on the bases of each participant’s body size and shape. Participants initially saw the avatar in the middle of this continuum and were specifically instructed to explore all the continuum before choosing the avatar’s body that best resembled their own in terms of shape and size. In this task, participants were standing up and the avatars were presented in a specular congruence with respect to their actual body, i.e., from a 3PP, as if they were looking at themselves in a mirror. The selected avatar (0% Avatar), the one 15% fatter (+15% Avatar) and the one 15% thinner (−15% Avatar) than the 0%, were used as virtual body stimuli for the embodiment blocks (Section 2.4.3).

#### 2.4.2. Perceived and Ideal Body Tasks: Body Dissatisfaction

**Figure 3:**
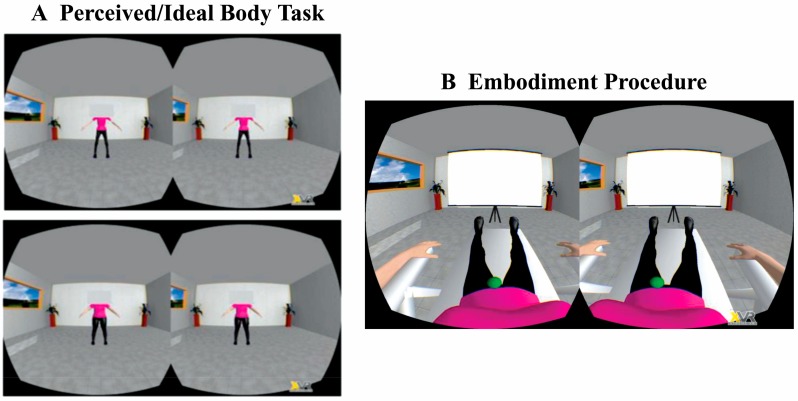
(**A**) Perceived/ideal body task. In separate blocks, participants choose the avatar which was the most similar to their own body (perceived body task) and the avatar which best resembled their ideal body (ideal body task) along a continuous of avatars presented from a third-person perspective (3PP). Each task comprised two trials presented in counterbalanced order: in one trial participants started the selection from the thinnest avatar (upper part of panel A), in the other from the fattest one (lower part of panel A). (**B**) Embodiment procedure. During the embodiment procedure a three minute of a/synchronous visuo-tactile stimulation was delivered. During the embodiment participants observed one of three different avatars from a first-person perspective (1PP). A virtual ball was programmed to touch the avatar on three different spots around the belly button in eight different ways (single touches and stroking movements).

To assess participants’ body dissatisfaction immediately before and after the IMS we asked them to choose the avatar which best resembled their real (perceived body task) and ideal (ideal body task) body in terms of size/shape/weight along the −30%–+50% continuum (Figure 3A). Differently from the avatar selection task, however, judgments were performed while participants were laying down on the deck chair and avatars were projected standing up in front of them, i.e., from a 3PP. As these tasks were performed before and immediately after IMS, participants were left lying on the desk chair to avoid disrupting any induced feelings of ownership over the avatar’s body.

Each task (perceived and ideal body task) comprised two trials, presented in counterbalanced order: in one trial participants started the selection from the thinnest avatar, in the other from the fattest one. Trials’ scores were then averaged for the final score. The discrepancy between the size of the ideal and the perceived body, calculated as the absolute difference between the two, was considered an index of participants’ body dissatisfaction.

#### 2.4.3. Embodiment Procedure

During the IMS procedure participants saw the body from a 1PP (Figure 3B) through a head-mounted display (Oculus Rift Developers Kit Dk1, Oculus VR, Menlo Park, CA, USA). Thus, the virtual body replaced the participant’s body in space. A calibration was performed to assure a proper positioning of the virtual camera and a precise overlap between the touch felt on the abdomen and the one observed on the avatar. Then, three minutes of visuo-tactile IMS were applied to the participant’s and the avatar’s body. The IMS was performed by a female experimenter, who received through headphones audio cues indicating the time and the location of each touch. 

For the experimental condition (Synchronous-IMS), we aimed to reach the maximal multisensory congruence between the real and the virtual body. Thus, in the synchronous condition the observed and felt touch matched in time and location, and we tracked participants’ head movements online to adjust visual perspective. However, as the visuo-proprioceptive congruence given by observing an avatar from a 1PP can be enough to induce feelings of ownership over a virtual body [43], we also aimed to reduce the possible occurrence of such illusory effects in the asynchronous control condition. Thus, we tried to boost the discrepancy in the control condition by delivering touches that were asynchronous in both time and location, as previous studies found that this stimulation was effective in maximizing the difference between synchronous and asynchronous conditions (see for example [44,45]) and we locked participant’s head tracking during the asynchronous IMS.

During visual-tactile stimulation, the participants were asked not to move their head and look at the belly that was stimulated. Before starting the stimulation, the experimenter made sure that the participant always looked at the virtual abdomen by continuously checking: (i) the orientation of the participants’ head (which had to be directed toward their real belly), and (ii) the virtual scenario on the PC monitor (where the virtual abdomen always had to be positioned in the center of the monitor).

#### 2.4.4. Explicit and Implicit Measures of Embodiment

As an explicit measure of embodiment, we used a self-reported questionnaire adapted from previous studies [19,33] assessing the strength of the illusion on three different components: Ownership, i.e., the sense of virtual body being one’s own; Agency i.e., the sense of being in control of the virtual body; and Referred Touch, i.e., the feeling of directly being touched by the seen ball (see Supplementary Materials for the complete list of items). As an implicit measure of embodiment, we recorded participants’ body temperature, taken through an infrared thermometer (IFR 100, Microlife AG, Widnau, CH, precision: ± 0.2 °C, 32.0–42.2 °C) under participants’ right armpit immediately after each block of IMS to compare ratings taken after synchronous vs. asynchronous embodiment blocks. Since we wanted to exclude participants with altered body temperature (due for example to febrile illness) we also took the body temperature before the experimental session started.

#### 2.4.5. Measure of Emotional Response Induced by Embodiment

Valence and intensity of the emotional response induced by being exposed to a/synchronous touching of the three differently sized avatars were assessed by a visual analogue scale (VAS) ranging from “very negative” (0) to “very positive” (100) presented after both synchronous and asynchronous IMS.

#### 2.4.6. Similarity and Attractiveness Ratings of the Avatars

As part of the final debriefing procedure, we checked how the −15%, 0%, and +15% avatars used during the embodiment blocks were actually perceived by the participants. Therefore, we asked participants to verbally rate on 0–100 VAS how much the three avatars resembled their own body (similarity ratings) and how attractive they thought these were (attractiveness ratings). The avatars were presented from a 1PP while participants were still laying down on the deck chair. Thus, ratings were collected while there was a spatial congruence between the actual participant’s body and the avatar’s body, i.e., while participants observed the three avatars replacing their own body in space. 

## 3. Results

Data were analyzed using STATISTICA version 8.0 (StatSoft, Tulsa, OK, USA). Significance was set at *p* < 0.05. The Duncan test was used for post-hoc comparisons. Bayes Factors were calculated by means the open-source software JASP [46] which allows quantification of evidence in favor of the alternative or null hypothesis. 

### 3.1. Baseline Measures

Descriptive statistics and independent sample *t*-tests were used for group comparisons of the demographical variables, eating disorder pathology and all the other baseline measures (Table 1).

Patients with anorexia nervosa (AN) reported higher symptoms severity scores in all scales (the Eating Disorder Inventory (EDI)—drive for thinness, EDI—body dissatisfaction scales, Body Shape Questionnaire (BSQ), Body Uneasiness Test—Global Severity Score (BUT GSI), but not at the EDI—bulimia, and at the Bulimic Investigatory Test, Edinburgh (BITE)). Patients with AN also had a lower body mass index (BMI) compared to healthy controls (HC). Body dissatisfaction (calculated as perceived minus ideal body) was higher in AN patients than in HC. While both groups were accurate and did not differ on the estimation of their perceived body, AN patients considered a thinner body as ideal compared to HC (see Table 2 for detailed statistics).

### 3.2. Explicit and Implicit Measures of Embodiment

Three separate 2 × 2 × 3 ANOVAs were run for each component (i.e., Ownership, Agency, and Referral of Touch) of the illusion, each with the factors Group, IMS, and Avatar. They revealed a main effect of IMS for the Ownership, Agency, and Referral of Touch (all Fs > 13.34; all ps < 0.001; all ηs2 > 0.259), suggesting a stronger illusion for synchronous as compared to the asynchronous IMS. We also found a main effect of Avatar on Ownership (F (1,38) = 7.85, *p* < 0.001, η2= 0.171)), with participants reporting higher scores for the +15% Avatar ((mean ± SE) (39.38 ±1.89)) compared to the 0% (33.78 ± 2.26) and to the −15% (32.08 ± 2.27) (all ps < 0.001). All the other main and interaction effects were not significant (all Fs< 3.35 all ps > 0.084).

The same 2 × 2 × 3 ANOVA run on the body temperature revealed a main effect of IMS (F (1,38) = 1.80, *p* = 0.002, η2 = 0.221)) showing a lower body temperature after the synchronous stimulation (34.91 ± 0.14) compared to the asynchronous one (35.02 ± 0.13). None of the other main and interaction effects were significant (all Fs < 1.27, all ps > 0.287). Please see Supplementary Materials for additional analyses.

These results suggest that there was no group dependent difference in how avatars were embodied and therefore a comparable level of bodily self-plasticity between AN and HC.

### 3.3. Body Dissatisfaction after Embodiment

A 2 × 2 × 3 ANOVA with Group (AN, HC) as between-and IMS (synchronous, asynchronous) and Avatar (−15%, 0%, +15%) as within-subjects factors showed no significant main or interaction effects (all Fs < 3.39, all ps > 0.073, all η2 < 0.065). Given that classical null hypothesis testing is not the ideal statistical tool for drawing conclusions about non-significant results [47,48], we also performed a Bayesian ANOVA which allows quantification of evidence in favor of the alternative or null hypothesis. The full model including main effects and the interaction between them provides evidence in favor of the null hypothesis (BF_10_ = 8.131 × 10^−5^), suggesting that embodiment of avatars of different body sizes did not change body dissatisfaction in AN and HC.

### 3.4. Emotional Response after Embodiment

**Figure 4:**
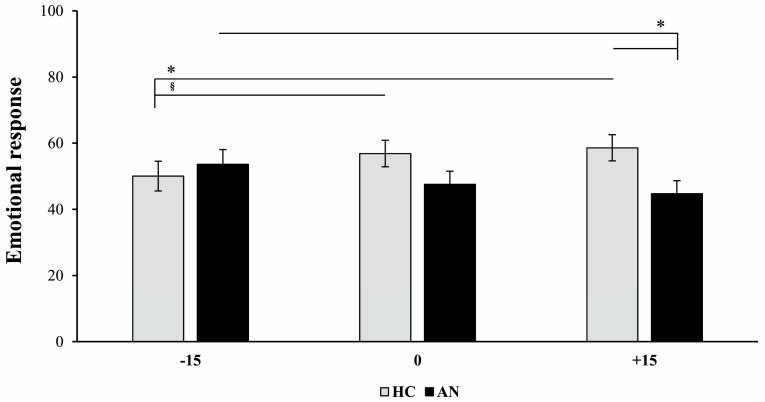
Emotional response after embodiment. Graph showing the effect of the interaction between avatar size (−15%; 0%; +15%) and group (healthy controls—HC; patients with anorexia nervosa—AN) on the emotional scale ranging from 0 (very negative emotions) to 100 (very positive emotions). Error bars represent standard error of mean. * = *p* < 0.05, § = marginally significant (*p* = 0.057).

The 2 × 2 × 3 ANOVA on the emotional ratings with the factors Group, IMS, and Avatar revealed a main effect of IMS (F (1,38) = 18.01, *p* < 0.001, η2 = 0.321), explained by more positive emotions following synchronous (59.44 ± 3.34) compared to the asynchronous (44.34 ± 2.93) IMS. The Avatar × Group interaction was also significant (F (2,76) = 7.21, *p* < 0.001, η2 = 0.159) (Figure 4) and shows that independently of the IMS, AN patients felt more negative emotions after being exposed to the +15% Avatar (44.71 ± 3.95) compared to the −15% Avatar (53.59 ± 4.50) (*p* = 0.017). The opposite trend was true for the HC who showed significantly more negative emotional response after being exposed to the −15% (50.02 ± 4.49) compared to the +15% (58.60 ± 3.95; *p* = 0.020) and marginally to the 0% Avatar (56.88 ± 3.99; *p* = 0.057). Finally, AN patients experienced more negative emotions to the +15% Avatar (44.71 ± 3.95) than HC (58.60 ± 3.95; *p* = 0.040). No other main or interaction effects were significant (all Fs < 3.73, all ps > 0.061). These results suggest that differently from the HC group, AN patients experienced negative emotions when they observed an avatar replacing their own body in space which reproduced a verisimilar increase in weight of 15%, with respect to the one that reproduced verisimilar decrease of weight of the same magnitude. This happened independently from the type of IMS used to induce the embodiment.

### 3.5. Avatars’ Similarity and Attractiveness Ratings

**Figure 5:**
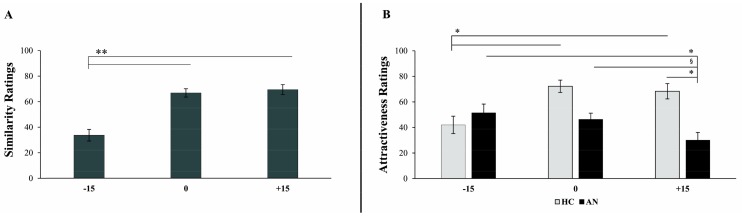
(**A**) Avatars’ similarity ratings. Graph showing the main effect of Avatar size (−15%; 0%; +15%) on similarity ratings given during the observation of the avatars from a 1PP. (**B**) Avatars’ attractiveness ratings. Graph showing the effect of the interaction between Avatar size (−15%; 0%; +15%) and Group (healthy controls, HC; patients with anorexia nervosa, AN) on attractiveness ratings given during the observation of the avatars from a 1PP. Error bars represent standard error of mean. ** = *p* < 0.001, * = *p* < 0.05, § = marginally significant, i.e., *p* = 0.058.

The 2 × 3 ANOVA with the factors Group and Avatar performed on the similarity ratings revealed a main effect of the Avatar (F (2,76) = 39.14, *p* < 0.001, η2 = 0.50): participants perceived the 0% (66.82 ± 3.26) and the +15% Avatar (69.37 ± 3.91) as more similar to their real body than the −15% Avatar (33.72 ± 4.52) (all ps < 0.001) (Figure 5, panel A). All the other main and interaction effects were not significant (all Fs < 0.59; all ps > 0.446). These results show that an increase in weight of 15% with respect to one’s own perceived body size might pass unobserved in both patients and controls, while a loss of weight of similar magnitude is detected.

The same ANOVA performed on the attractiveness ratings revealed a main effect of Group (F (1,38) = 12.07, *p* = 0.001, η2 = 0.241). HC rated the avatars as more attractive than AN (60.87 ± 3.71 vs. 42.63, ± 3.71). The Avatar × Group interaction was also significant (F (2,76) = 9.47, *p* < 0.001, η2 = 0.119) (Figure 5, panel B). AN considered the +15% Avatar as the least attractive ((30.10 ± 5.99) vs. the −15% Avatar (51.45 ± 6.83; *p* = 0.016) and the 0% Avatar (46.35 ± 4.82; *p* = 0.058. HC instead considered the −15% Avatar as the least attractive ((42.00 ± 6.83) vs. the 0% Avatar (72.25 ± 4.82; *p* < 0.001) and +15% Avatar (68.35 ± 5.99; *p* = 0.002)). The main effect of Avatar was not significant (F (2,76) = 2.74, *p* = 0.070). These results show that a loss of 15% of body weight is associated in AN patients to an increase in body attractiveness with respect to the perceived body weight (even though marginally) and to a verisimilar gain of the same magnitude in body weight, while it results in a decrease in body attractiveness with respect to the same categories of virtual bodies in HC participants.

### 3.6. Correlations between +15% Avatar Emotional Response and Symptoms Severity

**Figure 6:**
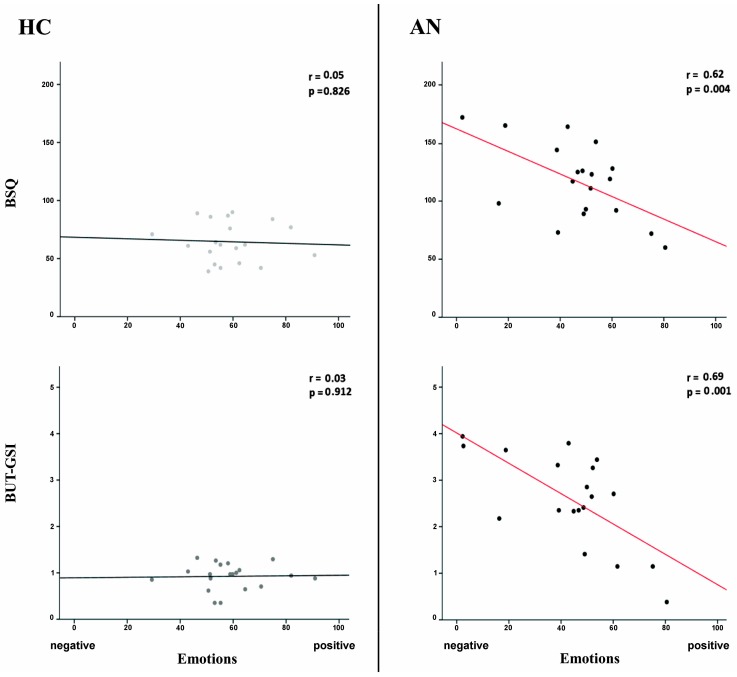
Correlations between +15% Avatar emotional response and symptoms severity. Scatterplots showing correlations between emotional changes after the exposure to the +15% Avatar and severity symptoms scores in the two groups. In the group of patient with anorexia nervosa (AN), the +15% Avatar emotional index correlated significantly with scores at the Body Shape Questionnaire (BSQ) and with the global severity index of the Body Uneasiness Test (BUT-GSI), while correlations were not significant in the healthy control (HC) group.

Finally, we tested, separately for each group, whether the emotions experienced with the exposure to the +15% Avatar (which was considered highly similar to the self and minimally attractive (Section 3.5)), was associated to self-reported body shape preoccupations, as indexed by the Body Shape Questionnaire (BSQ), and to the presence of abnormal body image concerns and eating behaviors, as indexed by the global severity index of the Body Uneasiness Test (BUT-GSI). We found that in AN patients, there was a significant correlation between the emotions experienced with the +15% Avatar and the strength of the concerns about the body shape (r = 0.62; *p* = 0.004; BF_10_= 14.05). Also, the correlation between the emotions experienced with the +15% Avatar and the BUT-GIS was significant (r = 0.69; *p* = 0.001; BF_10_ = 37.07). These correlations therefore suggested that the higher the symptoms’ severity was, the higher the negative emotional experience with the +15% Avatar (Figure 6, right panels). No significant correlation was found in the HC group (all rs < 0.05; all ps > 0.826; BFs_10_ < 0.283), Figure 6, left panels).

## 4. Discussion

We aimed to characterize, and eventually reduce, perceptual and cognitive-emotional components of body image distortion (BID) in AN using virtual bodies and embodiment illusion. To the best of our knowledge, only one study [49] investigated the body image in AN by: (i) using biometric self-avatars and (ii) reproducing the daily life experience of looking at oneself in the mirror. No studies instead coupled the creation of biometric self-avatars with multisensory bodily illusion paradigms.

Our results confirm that AN patients show higher body weight/shape concerns, drive for thinness (self-report questionnaires) and body dissatisfaction (perceptual minus ideal body size) compared to HC. However, body dissatisfaction in AN was not caused by a body overestimation, as suggested by previous literature [12]. Indeed, both AN patients and HC were accurate and did not differ in estimating the size of their real bodies, but AN patients desired a thinner body than HC. Although unexpected, these results are in line with a recent study [49] that adopted a virtual reality (VR) approach similar to the present one. Molbert and colleagues [49] measured body overestimation in AN by using a body scanner to create 3D avatars that faithfully reproduced participants’ real body and then manipulated these avatars to reproduce weight gains and losses. Analogously to our findings, results from this study show that AN patients perceived their body similar to the HC but, differently from them, they desired a thinner body. Thus, all together these results support the idea that BID in AN is characterized by distorted attitudes concerning the desired body rather than by perceptual overestimation of the body size. Moreover, in agreement with the results of a recent meta-analysis [14], our findings also support the idea that estimation of one’s own body size based on depictive methods (i.e., when participants estimate their body size by selecting a visual representation of their own body, like in this study) is less adapt to capture body size overestimation with respect to metric methods (i.e., when participants estimate their body size using quantifiable spatial estimations). This might be due to different features characterizing body representation that are targeted by these two types of methods. According to the body model proposed by Longo [50], the metric measures use both explicit and implicit knowledge of the body while the depictive measures, rely on explicit knowledge only, therefore they might be less automatic and more controllable.

The main aim of the present study was to investigate whether embodiment of differently sized avatars could reduce BID in AN. Therefore, we coupled virtual reality with a visuo-tactile IMS setup to induce embodiment of differently sized avatars, and measured embodiment strength and changes in perceptual and cognitive-emotional components of BID after embodiment induction. We adopted a controlled IMS procedure that differed from previous studies in several ways [15,28] and had the final aim of maximizing the congruence of virtual and real bodily signals. Thus we adopted both the 1PP and the head tracking during the synchronous IMS condition. This was particularly relevant in case of embodiment of bodies that patients might consider unattractive, as the ones reproducing their own weight and maximally in those reproducing a gain of 15% of this weight.

However, differently from [28], we used both synchronous and asynchronous IMS in order to disentangle the effects of embodiment and of observing the avatars from a 1PP. To this aim, we adopted a particular asynchronous control condition, in which we tried to maximize the incongruence between the virtual and real bodily signals. Indeed, the simple visuo-proprioceptive congruence between the real body and avatar’s body given by presenting the avatars from a 1PP might result per-se in illusory feelings of ownership of the observed body. Thus, to reduce the possible occurrence of such illusory effects also in the asynchronous control condition, we applied spatio-temporal asynchronous touches as in [44,45]. Also, we locked the head tracking of the virtual camera (differently from [15]), to reduce the visuo-motor congruency between the self and the virtual body and further disrupt possible illusory embodiment in the control condition (see Section 2.4.3 of the methods for details). Instead of using the same avatar size for all participants [28], we created customized avatars, matching actual weight, height, and body parts’ dimensions/shape and induced embodiment with the avatar reproducing participant’s perceived body size/shape and avatars reproducing realistic loss and gain in weight of 15% with respect to the perceived weight.

Both explicit (scores at the embodiment questionnaire) and implicit (body temperature data) measures of the embodiment suggest that our synchronous visuo-tactile stimulation was effective in inducing higher embodiment compared to the asynchronous one. Specifically, the answers at the self-report questionnaire show higher ratings after the synchronous compared to the asynchronous visuo-tactile stimulation for all the three components of corporal awareness. Participants were more likely to: (i) feel that the avatar’s body was their own one (Ownership), (ii) feel in control of its movements (Agency), and (iii) feel that the perceived touch was caused by the virtual one (Referral of Touch). These illusory sensations were independent of group and no interaction with the avatar size was found. Only for the Ownership component there was a main effect of the avatar size, with higher ratings attributed to the fatter avatars compared to both the 0% and the −15% avatar independently of group and type of IMS. Implicit measures of the embodiment mirror the explicit ones, as we found a change in body temperature between synchronous and asynchronous conditions independently of group and avatar size. However, the interpretation that this change in body temperature might be considered an implicit index of embodiment is currently highly debated in the literature [35] and we believe future studies are needed to clearly attribute the occurrence of such change to any evident factor (please see Supplementary Materials for a more detailed discussion on body temperature changes).

All in all, our measures on embodiment strength converge in showing that plasticity of body representation was similar in AN and HC. This result apparently contradicts previous literature which showed stronger bodily illusion for body parts (i.e., hands) in AN compared to HC participants [30]. It has been shown that bodily illusion negatively correlated with interoceptive abilities [29] and that higher bodily plasticity in AN plausibly results from altered multisensory integration of extero-and interocepetive signals [51]. In line with our results, HC and AN showed similar levels of embodiment of full bodies [31]. A conceivable reason for these contrasting results (rubber hand vs. full body illusion) might reside in the body part where touch is delivered. During the full body illusion touch is delivered to a highly salient and problematic body part for AN, i.e., the area around the abdomen. This may cause unpleasant sensations and negative emotions that in turn may dampen the embodiment in AN patients, making it similar to the level experienced by HC. Even though unpleasantness of the touch was not directly assessed in this study, AN patients anecdotally reported it.

An interesting finding of the present study is the fact that, independently of IMS type, AN patients showed more negative feelings after being exposed to the fatter avatar and that the strength of this effect correlated with clinical symptoms’ severity. HC, instead, showed more negative emotional reactions toward the thinner avatar, which were unrelated to body concerns and eating disorder measures. This is even more interesting when considering how much the three differently sized avatars were retrospectively considered physically attractive and similar to the self. Both AN and HC participants rated the perceived and the fatter avatars as most similar to themselves (compared to the −15%). However, AN patients found them to be the least attractive (and the −15% as the maximally attractive), while HC rated them as the most attractive (and the −15% as the minimally attractive). Thus, anorexics reacted negatively to fatter avatars which were considered highly self-resembling and less attractive. These results mirror results from a previous study in HC who embodied obese avatars (BMI of 32.3) observed from 1PP [52]. This experience increased body dissatisfaction and negative emotional reactions, and at a neural level changed activity of anterior cingulate cortex and anterior insula. Such regions are known to mediate negative body-related emotional and affective experiences, such as pain and disgust [53,54]. While these results may shed light on negative emotions experienced by anorexics, it is worth noticing that we did not include obese avatars. The personalized avatars increased by 15% were still below the over-weight range, considering the average BMI (18.98) in HC. We would like to notice that both in the synchronous and in the asynchronous IMS blocks the avatars were presented in the 1PP. As we reported above, simple visuo-proprioceptive congruence (1PP) may induce some illusory feelings of embodiment even during asynchronous visuo-tactile stimulation. Even though we introduced spatio-temporal incongruent touches and motor discrepancy to get illusory sensations in the asynchronous condition as low as possible, it is possible that such sensations were able to trigger an emotional response as in the synchronous condition.

Importantly, contrary to our predictions embodiment of differently sized avatars did not significantly change participants’ body dissatisfaction. This result stands in contrast with the results of a previous study by Preston and Ehrsson [28] conducted in HC only, which found that embodiment of a standardized slim body decreased body size perception and increased body satisfaction. Several differences may acknowledge for the discrepant results. Here, we measured body size perception with a task based on a visual representation of the body, i.e., participants had to estimate their body size on a customized avatar presented from a 3PP (body image). Instead, in the study by Preston and Ehrsson [28], perception of hip size was estimated by asking participants to indicate the distance on a ruler which reproduced their hip size in the absence of visual feedback (body schema). Participants were quite accurate in our body size estimation task, while participants in Preston and Ehrsson’s study [28] overestimated the size of their hips. In line with the above-mentioned discussion about the effectiveness of metric vs. depictive measures in detecting body size overestimation, these contrasting results suggest that IMS might be able to change body schema more than body image.

Absence of changes in body dissatisfaction might also be explained from a theoretical point of view by considering differences between the egocentric frame of reference [55,56], i.e., body perceived from the 1PP based on its present state constituted by interoceptive and exteroceptive inputs, vs. the allocentric frame of reference, i.e., a somatic representation of the body as a 3PP based on beliefs and attitudes related to the body. According to the Allocentric Lock Theory [57], people with AN are locked in their allocentric representation of the body and are unable to update it through egocentric sensory inputs. Indeed, in our study participants experienced embodiment of avatars of different sizes from an egocentric frame of reference, whereas the estimation of real/ideal body size was performed from an allocentric frame of reference. We can speculate that, even if the embodiment of differently sized avatars could have been successful in affecting the body image as experienced from an egocentric frame of reference, the inability for the AN patients to update their allocentric representation of the body through egocentric sensory inputs might have led to no changes in body dissatisfaction induced by the embodiment.

Related to the point above, our results also let us speculate that observing one’s own body from a 1PP (as it usually happens when we look down to our own body) or observing its reproduction from a 3PP (as it usually happens in front of a mirror, in pictures or videos) might bias our perception of its dimension. Indeed, when participants judge 3D reproduction of themselves without spatial or specular congruence with the self-body (as in the perceived body task), they are quite good at estimating their own body size. However, when they observe their own body by looking directly at it (as in similarity ratings task) they are more sensible to detect a loss than an increase in weight. Indeed, results from the similarity ratings show that an increase in weight of 15% with respect to one’s own perceived body size might pass unobserved in both patients and controls, while a loss of weight of similar magnitude is detected. This shows an asymmetry in how weight loss and gain might be considered by our perceptual system that seems to be detectable only when the to-be-judged body replaces our own in space. Also, our results suggest that the above-mentioned perceptual asymmetry is probably due to how we affectively experience our own body. Indeed, loss of weight is associated in controls to a decrease in body attractiveness, while in anorexic patients it is associated to an increase in body attractiveness. An increase of 15% in body weight is instead considered as attractive as the perceived body weight, both in patients and controls. If we focus on patients only, our results also suggest that when dealing with the affective component of the body, it does not matter whether one’s own body size is observed from a detached third-person perspective (ideal body task) or through directly looking at it (attractiveness ratings). One’s own perceived body size seem to be considered less desirable and attractive than a simulated loss of weight of 15%. On the same line, the simulated illusory experience of a gain in one’s own body weight is negatively experienced by anorexic patients, but not by controls (results from the emotional response task).

Overall, the present study suggests that the cognitive-emotional component of body image and not the perceptual one is severely altered in AN. Despite the inability to reduce body dissatisfaction in AN patients, our procedure was successful in inducing a strong embodiment of differently sized avatars, as measured at both the explicit and implicit level, and in enhancing negative emotional responses of anorexics to the fattest avatar which scaled with symptoms’ severity.

Future research and clinical trials should aim at changing the distorted cognitive-emotional components of body image through the internalization of a normal weight body and the reduction of the emotional distress caused by weight gain, more than at changing the perceptual ones. Additionally, even if one should be cautious in using stimuli of enlarged bodies, virtual reality could be used to gradually expose and habituate AN patients to healthier versions of their bodies and to act as an intermediary step prior to the in vivo body image exposure, as some therapeutic protocols are already showing (see [58] for a review).

## Figures and Tables

**Table 1:** Means (M) and standard deviations (SD) of demographic and eating disorders variables for the two groups (healthy controls-HC, and anorexics-AN), and results of the *t*-tests.

Demographic and Eating Disorder Variables							
	HC (N = 20)		AN (N = 20)				
	M	SD	M	SD	t	df	*p*
Age	23.85	3.23	23.30	7.60	0.29	38	0.767
BMI	18.94	0.98	15.86	1.12	9.22	38	0.001
EDI—drive for thinness	2.00	3.54	13.05	7.42	−6.01	38	0.001
EDI—bulimia	0.40	0.99	0.95	2.19	−1.02	38	0.313
EDI—body dissatisfaction	4.00	3.66	13.10	7.15	−5.07	38	0.001
BSQ	64.55	17.13	118.75	32.89	−6.54	38	0.001
BUT GSI	0.93	0.28	2.58	1.01	−7.07	37	0.001
BITE Symptoms	6.10	3.97	11.80	6.41	−3.38	38	0.002
BITE Severity	1.15	1.18	2.80	3.96	−1.79	38	0.082

BMI = Body Mass Index; EDI = Eating Disorder Inventory; BSQ = Body Shape Questionnaire; BUT GSI = Body Uneasiness Test, General Symptom Index subscale, BITE = Bulimic Investigatory Test, Edinburgh.

**Table 2:** Means (M) and standard deviations (SD) of perceived body, ideal body, and body dissatisfaction (perceived body minus ideal body) tasks measured at the baseline of the two groups (healthy controls-HC, and anorexic patients-AN), and results of the *t*-tests, i.e., *p*-values (p) and Bayesian factors (BF). Values are expressed as a % of the real bodies of the participants (100 is the real body size).

Perceived, Ideal and Body Dissatisfaction Measures								
	HC		AN					
Task	M	SD	M	SD	t	df	*p*	BF
Perceived Body	101.65	8.02	102.85	14.06	−0.33	38	0.742	0.32
Ideal Body	94.65	1.38	87.10	1.59	2.28	38	0.028	2.26
Body Dissatisfaction	8.45	7.85	19.95	12.97	−3.39	38	0.002	20.90

## Supplementary Materials

The following are available online at https://www.mdpi.com/2077-0383/9/1/98/s1.
