# Supplementary material for: Characterizing Body Image Distortion and Bodily Self-Plasticity in Anorexia Nervosa via Visuo-Tactile Stimulation in Virtual Reality
Source: J Clin Med. 2019 Dec 30;9(1):98. doi: 10.3390/jcm9010098 (PMC7019698; doi:10.3390/jcm9010098)
Supplement: Supplementary file 1 [file jcm-09-00098-s001.pdf]

Supplementary materials

Characterizing Body Image Distortion and Bodily Self-Plasticity in Anorexia Nervosa via Visuo-Tactile Stimulation in Virtual Reality

Luca Provenzano [1,2,+], Giuseppina Porciello [1,2,+,*], Sofia Ciccarone [1,2], Bigna Lenggenhager [3], Gaetano Tieri [2,4], Matteo Marucci [1,2,5], Federico Dazzi [6], Camillo Loriedo [7] and Ilaria Bufalari [2,8,*]

[1] Department of Psychology, "Sapienza" University of Rome, Italy;
[2] IRCCS, Santa Lucia Foundation, Rome, Italy;
[3] Department of Psychology, University of Zurich, Zurich, Switzerland;
[4] Virtual Reality Lab, University of Rome Unitelma-Sapienza, Rome, Italy, Italy;
[5] BrainTrends Ltd, Applied Neuroscience, Rome, Italy;
[6] Department of Human Sciences, Lumsa University, Rome, Italy;
[7] Clinica Psichiatrica, "Sapienza" University of Rome, Italy;
[8] Department of Developmental and Socialization processes, "Sapienza" University of Rome, Italy.
* These authors contributed equally to this work.

# Corresponding authors:
Prof. Bufalari Ilaria
Department of Psychology of Development and Socialization Processes
Sapienza University of Rome,
Via dei Marsi 78, 00185, Roma
E-mail: ilaria.bufalari@uniroma1.it

Or

Dr. Giuseppina Porciello
Department of Psychology
Sapienza University of Rome,
Via dei Marsi 78, 00185, Roma
E-mail: giuseppina.porciello@uniroma1.it

Supplementary Methods

S.1 Eating Disorder Questionnaires

The eating disorder inventory-2 (EDI-2) [1]. A 91-item self-report questionnaire measuring the clinical and psychological components of both anorexia and bulimia nervosa on a 6-point Likert scale. The test is organized into 12 primary scales: Drive for thinness, Bulimia, body dissatisfaction, low self-esteem, personal alienation, interpersonal insecurity, interpersonal alienation, interoceptive deficits, emotional dysregulation, perfectionism, asceticism, and maturity fears.

The body shape questionnaire (BSQ) [2]. A 34-item self-report questionnaires measuring the body shape preoccupations typical of bulimia nervosa and anorexia nervosa on a 6-point Likert scale.

The body uneasiness test (BUT) [3]. A 71-item self-report questionnaire that consists of two parts: BUT*A which measures weight phobia, body image concerns, avoidance, compulsive self-monitoring, detachment and estrangement feelings toward one's own body (depersonalization); and BUT*B which looks at specific worries about particular body parts or bodily behaviours. Items are answered on a 6-point Likert scale.

The Bulimic Investigatory Test, Edinburgh (BITE) [4]. A 33-item self-report questionnaire measuring bulimic behaviours and binge eating symptoms. The test consists of a symptom subscale that assesses the

presence of bulimic and binge eating symptoms and a severity subscale that indicates the severity of binge eating and purging behaviours based on frequency. Items are answered in a binary yes-no format.

S.2 Psychometric Questionnaire

The symptom checklist-90-R (SCL-90-R) [5] is a 90-item self-report questionnaire measuring nine primary symptoms dimensions: somatization, obsessive compulsive, interpersonal sensitivity; depression; anxiety; hostility; phobic anxiety; paranoid ideation and psychoticism. Additionally, SCL-90 provides three global indices of distress (global severity index, positive symptom distress index, positive symptom total). Items are answered on a 5-point Likert scale.

S.3 Self-Reported Embodiment Questionnaire

As an explicit measure of embodiment, we adapted a self-reported embodiment questionnaire (EQ) assessing participants' degree of explicit embodiment on three subscales: *Ownership, Agency, Referral of Touch* (adapted from previous studies e.g. [6,7]). The Ownership subscales refers to the illusion of feeling the virtual body as one's own. The Agency subscales refers to the sensation of being able to move and/or control the virtual body, whereas the Referral of Touch refers to the feeling of being touched by the virtual object. We also administered three control items to deviate participants' attention from the study hypothesis. Participants replied to the nine items by expressing their degree of agreement to each statement on a visual analogue scale (VAS) presented in VR ranging from "*completely agree*" (100) and "*completely disagree*" (0). The order of the items (see Table 1) was randomized.

Table 1. Embodiment questionnaire items assessing participants' degree of explicit embodiment on four subscales.

Embodiment Questionnaire Items	
**Items**	**Subscales**
I felt I was looking at my own body	Ownership
I felt the virtual body was my own body	Ownership
I felt I could control the virtual body	Agency
I felt I could move the virtual body	Agency
I felt the virtual body obeyed or could obey my will	Agency
I felt that the touch I received was caused by the ball moving on the virtual body	Referred Touch
I felt I was receiving the touch where the ball was touching the virtual body	Referred Touch
I felt I had more than one body	Control
I felt I did not own a body anymore	Control

Supplementary Results

Body Temperature Changes

As we stated on the main text, as an implicit measure of embodiment, we recorded participants' body temperature, taken through an infrared thermometer (IFR 100, microlife, precision: ± 0.2°C, 32.0 ~ 42.2 °C) under participants' right armpit, at baseline and immediately after each block of IMS. Since we wanted to exclude participants with altered body temperature (due for example to febrile illness) we also took the body temperature before the experimental session started.

Results from the 2 x 2 x 3 ANOVA run on the body temperature revealed a main effect of IMS [$F(1,38) = 1.80$, $p = 0.002$, $\eta2 = 0.221$)] showing a lower body temperature after the synchronous stimulation (34.91 ± 0.14) compared to the asynchronous one (35.02 ± 0.13). None of the other main and interaction effects were significant (all $Fs < 1.27$, all $ps > 0.287$). It is important to say that even if our thermometer had a precision of ±0.2 °C, the error interested all the conditions in the same measure. However, to be more conservative and check that our results were not influenced by the measurement error, we performed an additional analysis where we subtracted the temperature measured in the synchronous conditions from those measured in the asynchronous ones and derived an index of the effect. We then removed all the differences that were lower than ±0.2 °C and performed a t-test against zero (i.e., no differences between synchronous and asynchronous

conditions). Results from the t-test against zero ($t(10) = 4.998$; $p = 0.0005$) supported our original finding, i.e., the two conditions significantly differed.

Supplementary Discussion

The fact that changes in body temperature might be interpreted as an implicit index of embodiment is currently highly debated [8] in the literature. Indeed, some studies reported body temperature decreases of the real body while others reported body temperature increases, and at present, direction of body temperature changes occurring during embodiment is not clearly attributable to any factor [8]. However, we think that our results on temperature might be different from the results by [9,10] where decrease in body temperature/pain perception was interpreted as a phenomenon originated from the dis-ownership of the real hand in favor of the embodied one (the rubber hand). Indeed supporting the idea by Tieri et al. [11], our data show that when a 1PP is used for creating the full body illusion in virtual reality, participants' real body representation is replaced by the virtual body and no dis-ownership feelings are generated. More evidence is needed to better understand how body temperature is modulated by embodiment feelings and probably more sophisticated instruments are needed.

S.4 Bibliography

1. Garner, D. *Eating Disorder Inventory-2: Professional manual*; Psychological Assessment Resources Inc: Odessa, FL, 1991;
2. Cooper, P.; Ph, D.; Taylor, M.; Sc, B.; Cooper, Z.; Ph, D.; Fairburn, C.G. The Development and Validation of the Body Shape Questionnaire. *Int. J. Eat. Disord.* **1987**, *6*, 485–494.
3. Cuzzolaro, M.; Vetrone, G.; Marano, G.; Garfinkel, P.E. The Body Uneasiness Test (BUT): Development and validation of a new body image assessment scale. *Eat. Weight Disord.* **2006**, *11*, 1–13.
4. Henderson, M.; Freeman, C.P.L. A Self-rating Scale for Bulimia The "BITE." *Br. J. Psychiatry* **1987**, *150*, 18–24.
5. Derogatis, L.R. Symptom Checklist-90-R: Administration, Scoring & Procedure Manual for the Revised Version of the SCL-90; National Computer Systems: Minneapolis, 1994;
6. Lenggenhager, B.; Tadi, T.; Metzinger, T.; Blanke, O. Video ergo sum: Manipulating bodily self-consciousness. *Science (80-.).* **2007**, *317*, 1096–1099.
7. Tieri, G.; Tidoni, E.; Pavone, E.F.; Aglioti, S.M. Body visual discontinuity affects feeling of ownership and skin conductance responses. *Sci. Rep.* **2015**, *5*, 17139.
8. de Haan, A.M.; Van Stralen, H.E.; Smit, M.; Keizer, A.; Van der Stigchel, S.; Dijkerman, H.C. No consistent cooling of the real hand in the rubber hand illusion. *Acta Psychol. (Amst).* **2017**, *179*, 68–77.
9. Mancini, F.; Longo, M.R.; Kammers, M.P.M.; Haggard, P. Visual distortion of body size modulates pain perception. *Psychol. Sci.* **2011**, *22*, 325–330.
10. Moseley, G.L.; Olthof, N.; Venema, A.; Don, S.; Wijers, M.; Gallace, A.; Spence, C. Psychologically induced cooling of a specific body part caused by the illusory ownership of an artificial counterpart. *Proc. Natl. Acad. Sci.* **2008**, *105*, 13169–13173.
11. Llobera, J.; Sanchez-Vives, M. V.; Slater, M. The relationship between virtual body ownership and temperature sensitivity. *J. R. Soc. Interface* **2013**, *10*.
